# Economic Conditions of Young Adults Before and After the Great Recession

**DOI:** 10.1007/s10834-017-9554-3

**Published:** 2017-10-17

**Authors:** Maria Sironi

**Affiliations:** Department of Social Science, University College London, 55-59 Gordon Square, London, WC1H 0NU UK

**Keywords:** Transition to adulthood, Employment, Economic conditions, Great recession, Luxembourg income study

## Abstract

Transition to adulthood has undoubtedly changed in the last few decades. For youth today, an important marker of adulthood is self-actualization in their professional career, and, consequently, also the achievement of stable financial conditions. Economic conditions of youth are greatly subject to fluctuations in the economy, and the subsequent governmental response. Using the Luxembourg Income Study, this work investigates the trends in income from work of young adults before and after the Great Recession of 2008 in five countries—US, UK, Norway, Germany, and Spain. The findings showed deterioration in economic conditions of young men, but with differences across countries. Young women suffered less from the crisis, and in some countries, their economic situation improved. The general negative trend was especially pronounced for those with high education, which is primarily because they stayed in education longer.

## Introduction

In western societies, the way in which adolescents transition to adulthood has transformed in the last decades. In the 1950s and 1960s, young people faced key events, such as starting to work full-time, getting married, and having children, by their early twenties. Today, those roles are postponed to the late twenties and early thirties (Aassve et al. 2002; Furstenberg 2010; Settersten et al. 2008; Sironi and Furstenberg 2012).

In addition to the slower path to adulthood, the trajectories became more diverse (Rindfuss 1991). The standard sequence of events leading into adulthood in the 1950s and 1960s—graduate from school, move out from the parental home, start working full-time, marry, have children—changed. The order of events became less linear and it became more likely for youth to experience multiple events at different points in time (Elzinga and Liefbroer 2007; Marini 1984a, b). Several studies document the variation in major steps of the transition to adulthood, including the school-to-work transition, the expansion of education (Blanchflower and Freeman 2000; Gangl 2002; Quintini et al. 2007), and the departure of young adults from the parental home (Mandic 2008; Mulder and Clark 2000; Mulder et al. 2002).

The recent economic recession has had important implications for young adults’ lives. Several studies documented how the economic crisis hit youth the hardest, as they experienced the largest increase in unemployment rates, which persisted long after the crisis (Grusky et al. 2011; Hout et al. 2011; Mínguez et al. 2012; O’Higgins 2014; Verick 2009). Further, others investigated the impact of the recession on fertility, partnership formation and dissolution (Cherlin et al. 2013; Sobotka et al. 2011). However, only a few studies focused on how youths’ economic conditions and wages changed over time and across countries (Bell et al. 2007; Smeeding and Phillips 2002), and in particular, on how the Great Recession affected them. This paper aims to aid in filling in this gap in the literature.

In the past, adulthood was associated more with family formation, but today, it is associated more with self-actualization in education and career, and, consequently, also with financial conditions (Berlin et al. 2010; Furstenberg et al. 2003). With this shift in mind, it is increasingly relevant to look at how the recent economic and financial crisis affected the economic standing of young adults. The questions this work attempts to explore are: How did the economic position of young adults change over time and across countries? What are the consequences of economic uncertainty and instability on income from work? What groups have suffered the most from the Great Recession?

This study describes how the employment and economic conditions of young adults have changed since the beginning of the new century in Westerns societies, and it looks at trends before and after the economic crisis. Given the differing timing of the transition to adulthood in different parts of the world (Billari 2004; Billari and Liefbroer 2010; Liefbroer and Goldscheider 2006) and the different implications of the economic crisis in different countries, this work adopts a cross-national comparative perspective. The national context likely influenced the way in which the crisis developed and which economic sectors or groups of the population the recession affected the most. Different countries have very different welfare regimes, culture, demographic trends, and labor market structure, and these characteristics might have had an impact on how the recession was handled by the governments, absorbed by the labor market and perceived by different sociodemographic groups.

Hence, it is necessary to consider a sample of diverse experiences to detect common and divergent trends. Using data from the Luxembourg Income Study of 1999/2000, 2004, 2007 and 2010, this work compares young adults in five countries: US, UK, Norway, Germany, and Spain. Documenting and analyzing changes in the economic conditions of young adults before and after the crisis may help to understand their subsequent life trajectories, such as partnership and fertility histories, and to identify—or even construct—social policies that are adequate to support them.

## Economic Position as Part of the Transition to Adulthood

### Changes in the Transition to Adulthood

The transition to adulthood today is very different than it was right after World War II: It starts later, it takes longer, and it happens in a less standardized fashion (Buchmann and Kriesi 2011; Sironi 2015). There was a significant expansion of education in developed countries in the last 60 years. The median age at leaving school went up, which also caused a delay in the age at first full-time employment (Corijn and Klijzing 2001; Schizzerotto and Lucchini 2004). For example, people born in the 1950s in the UK, Italy and Germany completed school when they were on average 16, while in 2011, the median age of completing school increased to 20–22 (Buchmann and Kriesi 2011; Schizzerotto and Lucchini 2004). In the US, 90 and 43% of the people born in the late 1970s and early 1980s attained upper secondary and tertiary education, respectively (OECD 2005). In the UK and Scandinavian countries, 50% attained tertiary education in 2012. The lowest rates were found in Germany and southern European countries (OECD 2009), which were below 30%.

Moreover, the labor market structure evolved greatly, with more instability and greater difficulties in getting a job (Bernardi et al. 2004; Blanchflower and Freeman 2000; Breen 2005; Brzinsky-Fay 2007, 2008; Gangl 2002; Ryan 2001; Scherer 2005). Since the 1970s, earnings inequality went up, and job tenure declined (Duncan et al. 1996; Katz 1999; Sironi and Furstenberg 2012). The job path did not only change over time, but it also showed some differences by region and labor market structures. When Southern Europeans complete their educational career, they very frequently experience a period of instability, with several temporary contracts. Moreover, youth unemployment is currently highest in Southern Europe (Brzinsky-Fay 2007; Quintini et al. 2007). The situation is different in continental countries, with a smoother transition into the job market (Gangl 2002; Quintini et al. 2007; Scherer 2001). In fact, young adults in those countries face high uncertainty after they exit education, but only for a short time.

The trends mentioned so far are among the most well-known and well-studied regarding the transition to adulthood related to economic conditions. However, one less investigated—but fundamental—trend leading to adulthood is the economic circumstances of young adults. Smeeding and Phillips (2002) analyzed young people’s earnings in the 1990s in seven countries (France, Germany, Italy, Sweden, UK, US, and the Netherlands) using the Luxembourg Income Study for just one point in time. They measured economic conditions in many different ways: They looked at full-time employment, wage earned, earnings inequality, social transfers, and family resources. They found that in the mid-1990s, only a small percentage of young adults were financially self-sufficient (based on different indicators such as working full-time, earnings above the poverty line for a single-person household, and earnings above the poverty line for a three-person household) in their early twenties. This situation persisted even when welfare transfers were considered (Aassve et al. 2006).

Bell et al. (2007), on the other hand, compared trends in six countries (US, UK, Canada, Belgium, Italy, and Germany) on household living arrangements, employment rates, and earnings levels in the mid-1980s and late 1990s, as youth aged from eighteen to thirty-four. Their results showed decreasing trends in economic self-sufficiency (i.e., young adults’ earning more than the median adjusted disposable net household income in their country or earnings above the national poverty line in a given year) over time, except for women in their late twenties and early thirties, even though they still earned less than men. The US, and to some extent the UK, were exceptions to this general pattern. Here, the employment rate improved for young adults, and wages stayed stable or increased slightly (Bell et al. 2007).

### Implications of the Great Recession on Youth

The economic crisis that started in 2008 and affected most developed economies had its largest impact on young adults. Many young people worked in sectors that were particularly affected by the recession, such as manufacturing, construction, services and tourism (O’Higgins 2014; Verick 2009).

Youth were generally more vulnerable to unemployment because there was a greater risk of losing their jobs and an increased difficulty to enter the labor market (Marcus and Gavrilovic 2010). In 2009, 40% of the unemployment segment of the population was between 15 and 24 years old (O’Higgins 2014), and the global youth unemployment rate rose from 11.9 to 13% between 2007 and 2009. The 1% increase in youth unemployment rate between 2008 and 2009 corresponded to the 0.5% increase in the adults’ rate. In developed societies, the youth unemployment rate went up by 4.6% between 2008 and 2009, reaching a rate of 17.7% in 2009 (ILO 2010).

The proportion of employed young women was lower than that of young men, but most regions showed decreasing gaps between male and female labor force participation rates. In the US, where the crisis started, the youth unemployment rate among men rose from 12.1% in 2008 to 19.8% in 2009, while the increase for young women was 5.9%. Similar figures were observed in the UK, with an increase from 15.4 to 21.8% for young men, and from 11.9 to 16.2% among young women. The increase in Norway and Germany was more modest, with a 2.7% increase for men and a 0.9% increase for women in Germany, and a 2.1% increase for men and a 1.8% one for women in Norway. Spain showed a rise of 21.1 and 11.5% for men and women, respectively (Verick 2009).

Youth unemployment rate differentials not only change by country and gender, but also by education levels. The crisis hit more young people with higher (tertiary) levels of education (O’Higgins 2014).

These trends had negative consequences in the short-run and in the long run related to the careers of young adults and their unemployment. Most likely, these individuals did not have financial resources that could support them during unemployment spells, and very often, they had to rely on their family/parental income. Moreover, among the young people who could find a job during the recession, working conditions were possibly poor; they were forced to work part-time, with informal and temporary contracts, which might have resulted in low wages and little social protection. This work focuses on employment outcomes of young adults and, in particular, on income from work to try and capture a more comprehensive picture of their working conditions before and after the crisis.

### Differences in Trends Across Countries

The evidence of a convergence in life course pathways in different western societies is scarce (Billari and Wilson 2001; Liefbroer and Goldscheider 2006). If anything, the clearest trend is that of *converging divergences* (Blossfeld et al. 2005; Mills et al. 2008), meaning that most countries have shown growing diversity at the individual level (Buchmann 1989; Kohli 2007; Shanahan 2000). There are three possible reasons behind this variation in individual behavior: the difference in welfare regimes across countries, the way in which labor markets are regulated, and different cultures. Individual life course is clearly influenced by these three aspects, which create opportunities and limitations to which young adults have to adjust (Breen and Buchmann 2002; Sironi 2015).

If we consider welfare regimes, for example, and their possible influence on individual behavior, we see how each regime is linked to a different pathways to adulthood (Buchmann and Kriesi 2011; Esping-Andersen 1990; Mayer 2001; Sironi 2015). In *Social-democratic* countries (e.g., Northern European countries), that have very generous welfare regimes, young adults leave their family of origin early, and also start working relatively early. For countries with a *conservative* welfare state (e.g., France, Germany, and the Netherlands), which is characterized by generous support for the family, or for those with a *liberal* welfare regime (e.g., the UK and the US), which provides only means-tested benefits, the standard life course pathway is more difficult to predict; however, it should be less de-standardized than in Northern Europe. *Southern European* countries have the family as the main provider of support, since the welfare provisions are not substantial (Ferrera 1996; Mayer 2001; Trifiletti 1999); thus, young adults in these countries leave their parents late, marry late and have children late (or at least later than in other European countries) (Aassve et al. 2002, 2006; Aassve and Lappegård 2009; Billari 2004; Billari et al. 2001; Esping-Andersen 1990; Mills et al. 2008; Mulder et al. 2002).

Welfare regimes are very closely interrelated with the cultural characteristics of each country. In *conservative* regimes the welfare transfers are given only to the household head. This system incorporates a male-breadwinner and hierarchical family model, in which women and children are considered as dependents (Breen and Buchmann 2002). The same is true in *Southern European* countries, but mainly because of scarce welfare provisions. This cultural system of a male-dominated society reinforces the way and the timing in which both women and youth enter the labor market. Even with substantial changes over time, the female labor force participation remains lower than for men, and young adults have temporary or precarious jobs for longer time. The opposite situation occurs in *Social-democratic* countries, where the egalitarian culture promotes gender equality, and where young adults are considered as independent and responsible individuals (Buchmann 2002). Hence, not only because of generous welfare transfers, but also because of the cultural perception of young adults as active and autonomous members of the society, youth enters the labor market and separates from the family of origin very early. Finally, the *liberal* welfare regime supports an individualistic and competitive culture, in which young adults are also independent individuals, even though their choices and behavior can have riskier consequences due to a less generous welfare system.

These differences in welfare states, and the varying cultural and institutional aspects across countries, imply that the impact of the economic crisis was different in different settings (Blossfeld et al. 2005). Given the unstable opportunity structure associated with the recession, and the deterioration in employment and earnings levels, young people likely adapted their behavior to improve their immediate and short-term living conditions. Consequently, their choices reflected the specific combination of the opportunities in the labor market, welfare state provisions, and family support in their country of residence (Vogel 2002).

Given the differing paths into adulthood and the timing of the transition in different parts of the world (Billari 2004; Billari and Liefbroer 2010; Liefbroer and Goldscheider 2006), and also the different implications of the Great Recession for young adults’ economic standing, this work adopts a cross-national comparative perspective. It compares young adults in the US, the UK, Norway, Germany, and Spain in 2000, 2004, 2007 and 2010. More specifically, it looks at the proportion of young adults between age 22 and 30 who are working full-time and who are *low-paid* (more on the definition of *low-paid* workers in the next section), and at the factors associated with their economic conditions, in particular education.

## Data and Methods

### Data

The Luxembourg Income Study (LIS) contains harmonized microdata from high- and middle-income countries around the world beginning in the late 1960s and early 1970s. National surveys were harmonized to create databases that are comparable and that allow researchers to examine similarities and differences across countries. LIS includes information on employment status, paid hours of work, and income. The analysis presented here examined data at four points in time (2000,^1^ 2004, 2007, 2010) in five countries, representative of the different patterns in the transition to adulthood and different typologies of welfare states: the US and the UK (representing the liberal welfare state), Norway (representing social-democratic welfare state), Germany (representing conservative welfare state), and Spain (representing Southern Europe).

The use of LIS had some limitations. First, given the harmonization process, some pieces of information included in the original data sets were lost, and not all the countries and all the years included the same variables. Also, sample sizes varied across countries, which affected reliability of the estimates in the countries with a smaller number of observations and affected the regression results on the pooled data (driven by the countries with the largest sample size). Despite these limitations, harmonized data in the LIS were unique because they included information on labor earnings for all countries and did not suffer from problems of missing values. There were no other data sources comparing different developed countries with information on individual earnings that were harmonized and that started early enough to compare young adults economic conditions over time. The sample used in this work included individuals between age twenty-two and thirty at different points in time in order to observe changes before and after the crisis, but to also take into account continuing trends that were taking place before the onset of the crisis.

### Methods

To show a comprehensive picture of young adults’ working conditions, the first step was to investigate the proportion of youth working full-time. Following the definition used by Smeeding and Phillips (2002), *working full-time* was defined as working more than 35 h per week and more than 40 weeks per year.^2^

The second step was to look at the economic conditions of young adults in the sample. To do that, the OECD definition of *low-paid* workers was used, and the proportion of young adults who were *low-paid* was computed. *Low-paid* young adults were those earning wages less than two-thirds of median earnings. Therefore, the measure of earnings was constructed by computing the individual median income in each sample among those who were employed. The median income for youth in each country and in each time period is shown in Table 1. This measure gives only a partial picture of the financial status of young adults. However, it does give a sense of the level of wages in each country, by age and sex, and shows changes in trends over time.

**Table 1:** Median income and *Low-paid* income

	Median income of those with a job	Currency	Low paid income	Exchange Rate to USD	Low paid income in USD
US					
2000	21,000	USD	14,000.0	1.00	14,000.0
2004	22,000	USD	14,666.7	1.00	14,666.7
2007	25,000	USD	16,666.7	1.00	16,666.7
2010	25,000	USD	16,666.7	1.00	16,666.7
UK					
1999	13,277	GBP	8851.3	0.62	14,276.3
2004	15,432	GBP	10,288.0	0.55	18,705.5
2007	16,800	GBP	11,199.7	0.50	22,399.3
2010	16,640	GBP	11,093.3	0.65	17,066.7
Norway					
2000	213,562	NOK	142,374.7	8.80	16,178.9
2004	234,077	NOK	156,051.0	6.74	23,153.0
2007	281,576	NOK	187,717.3	5.86	32,033.7
2010	308,983	NOK	205,988.7	6.04	34,104.1
Germany					
2000	32,500	DEM	21,666.7	1.80	12,035.0
2004	14,426	EUR	9,617.3	0.80	11,963.0
2007	18,255	EUR	12,170.0	0.73	16,679.0
2010	17,975	EUR	11,983.3	0.75	15,886.3
Spain					
2000	1,362,498	ESP	908,331.7	153.12	5,932.3
2004	9960.0	EUR	6,640.0	0.80	8,259.5
2007	13,508.0	EUR	9,005.3	0.73	12,341.8
2010	13,680.0	EUR	9,120.0	0.75	12,090.4

*Low-Paid* income corresponds to two-thirds of the median income

Both measures were calculated using person weights included in the LIS to account for the sampling probability and the different age structures in different countries. Given gender differences in paths into adulthood and different labor force participation rates, the analyses were performed separately for men and women.

Next, several logistic regressions were performed to study the association between economic conditions of young adults and different individual and contextual characteristics: The first specification looked at the association between being *low-paid* and age, gender, year, and country of residence. Then, to explore possible differing trends between men and women over time, an interaction term between *gender and year* was added in the second specification. In the third specification, an interaction term between *year and country variables* was included, given the possibility that trends over time and the impact of the crisis differed across countries. The individuals’ *level of education* 3 was introduced in the fourth model. Past evidence showed that across all developed societies, young people from lower social classes usually leave school and start working earlier (Bynner 2005; Muller and Shavit 1998). Hence, it is possible that they achieved a good financial position earlier. However, low levels of education are usually associated with unstable employment (Arts and Gelissen 2002; Blossfeld et al. 2005; Furstenberg 2008), thus increasing the risk of future poverty. Moreover, it is possible that the crisis affected different levels of education in different ways, therefore, an interaction term between *year and education level* was included in the fifth specification.

Finally, since the crisis hit more young people with higher levels of education (O’Higgins 2014), the possibility that the crisis and the lack of jobs pushed young adults to stay in school longer was investigated. To do that, the fifth specification described above was repeated including the variable *enrollment in school* (equal to 1 if still in school, and 0 otherwise). This information on enrollment in school was not included in the 2000, 2004, and 2010 data for Norway. Hence, this last set of analyses was performed only on the US, UK, Germany, and Spain. If the new predicted probabilities showed a flatter trend over time when *enrollment in school* was taken into account, especially for those with high education, it would be an indication that they decided to prolong education and postpone the entry into the labor market—consequently postponing the onset of earning wages.

## Results

### Descriptive Findings

Table 2 presents characteristics of the selected samples, by country and survey year. The mean age in the sample was approximately twenty-six in all countries, and the proportion of women ranged between 48.1% (Norway in 2004) and 56.2% (Germany in 2007). As expected, the proportion of people reporting a high level of education increased over time, except in Norway, where the proportion with a tertiary education was already high in 2000 (31.3%). The greatest increase was observed in the UK, where 23.7% of the 2000 sample completed tertiary education, rising to 34.5% in 2007 and 32.8% in 2010. The smallest proportion of people with high education in 2010 was reported in Germany (19.2%). Differences across countries could be due to the fact that the expansion of education unfolded in different ways across countries, both its starting point and the rapidity of expansion. Therefore, educational systems were not homogeneous. These differences should be considered when interpreting the results.

**Table 2:** Descriptive statistics—weighted by country and year Source: UNECE statistical division database

Country and year	Mean age	% Female	% In school	% With high education	% Working full-time	Unemployment rate (%)	N	National data source
US								
2000	26.1	51.0	6.2	34.7	66.3	4.0	22,441	CPS
2004	25.9	50.8	7.5	33.7	61.0	5.5	21,093	
2007	26.0	50.2	7.8	36.6	62.4	4.6	21,280	
2010	26.0	49.9	8.6	38.7	54.1	9.6	21,550	
UK								
1999	26.2	49.2	14.3	23.7	63.9	5.9	6038	Family expenditure survey
2004	26.0	50.9	17.4	30.1	63.8	4.7	6194	
2007	25.9	50.3	8.0	34.5	63.3	5.3	5397	
2010	26.0	50.1	10.3	32.8	55.3	7.8	5619	
Norway								
2000	26.3	49.0	–	31.3	72.3	3.2	3804	Income distribution survey
2004	26.2	48.1	–	28.2	68.9	4.3	3485	
2007	26.1	49.3	23.5	32.6	69.0	2.5	52,725	Household income statistics
2010	26.0	49.0	–	31.8	64.2	3.6	56,141	
Germany								
2000	26.2	51.5	26.5	16.5	59.7	8.0	2733	GSOEP
2004	26.0	53.6	29.0	12.8	53.7	10.5	2454	
2007	26.3	56.2	21.9	18.9	56.6	8.7	1921	
2010	26.0	55.4	28.6	19.2	56.2	7.1	1997	
Spain								
2000	26.1	51.1	18.5	24.2	55.1	11.9	2242	ES ECHP
2004	26.3	49.0	19.6	32.8	61.7	11	4386	EU-SILC
2007	26.3	48.9	21.6	25.7	61.1	8.2	3903	
2010	26.3	49.7	26.4	25.9	44.6	19.9	3274	

For Norway the % working full-time reflects only those who state to be employed (no info on hours and weeks of work)

As shown in Table 2, the percentage working full-time decreased over time in each country. The magnitude of the change was, however, quite diverse across countries. In the US, UK, and Norway, there was a small decrease from 2000 to 2007, with a larger drop between 2007 and 2010, which was most likely due to the hit of the crisis.

In Germany, there was a very small decline (3%) between 2000 and 2007, but there was almost no change between 2007 and 2010. In Spain, the trend in the percentage of young adults employed full-time was positive from 2000 to 2007, but this figure dropped drastically in 2010 (from 61.1 to 44.6%).

Macro-level employment rates showed almost no change in Germany and Norway between 2000 and 2010, with some fluctuations within the two periods. Both the US and the UK reported an increase in the unemployment rate, from 4 to 9.6% and from 5.9 to 7.8%, respectively. Coherent with the micro-level statistics, Spain was the country that showed the largest deterioration: The unemployment rate went from 11.9% in 2000 to 19.9% in 2010, which was even worse if we consider that the rate was 8.2 in 2007. It is worth noticing that the unemployment rate referred to the entire population in working age, and not only to the age range (22–30) considered in the analysis.

### Employment Trends and Economic Conditions by Gender

**Fig. 1:**
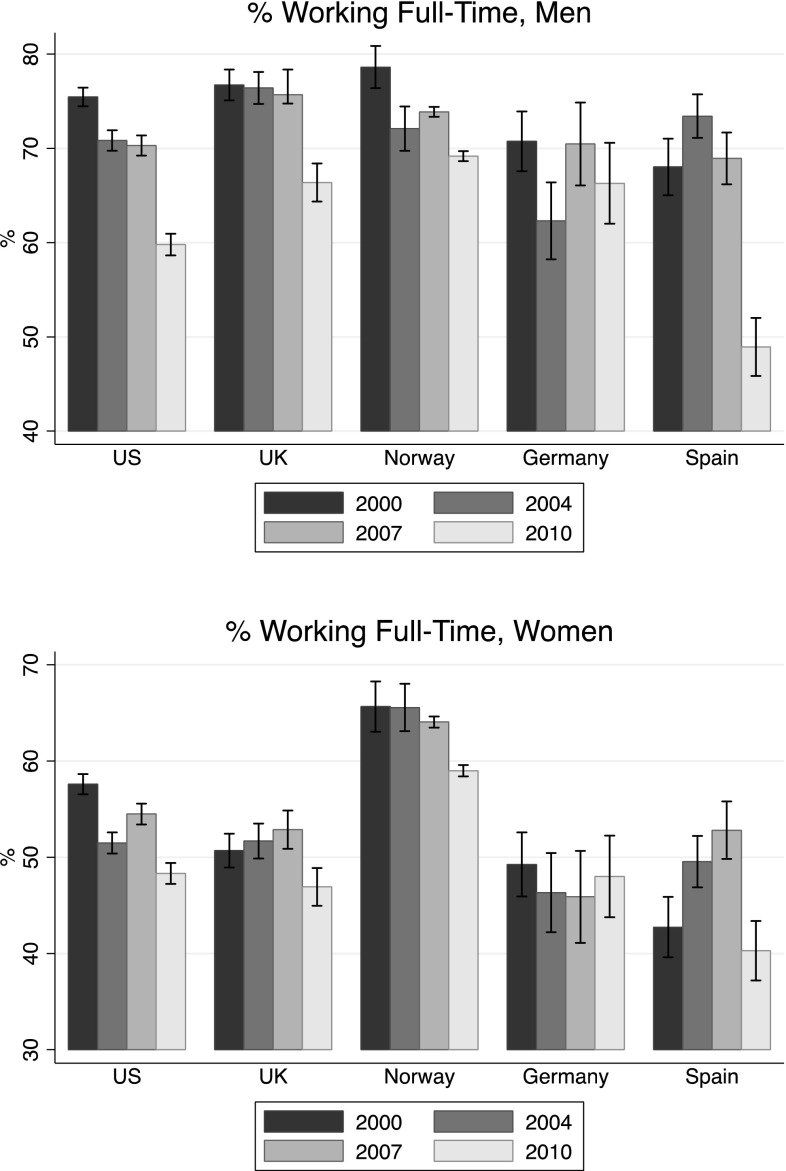
% of Men and women working full-time

**Fig. 2:**
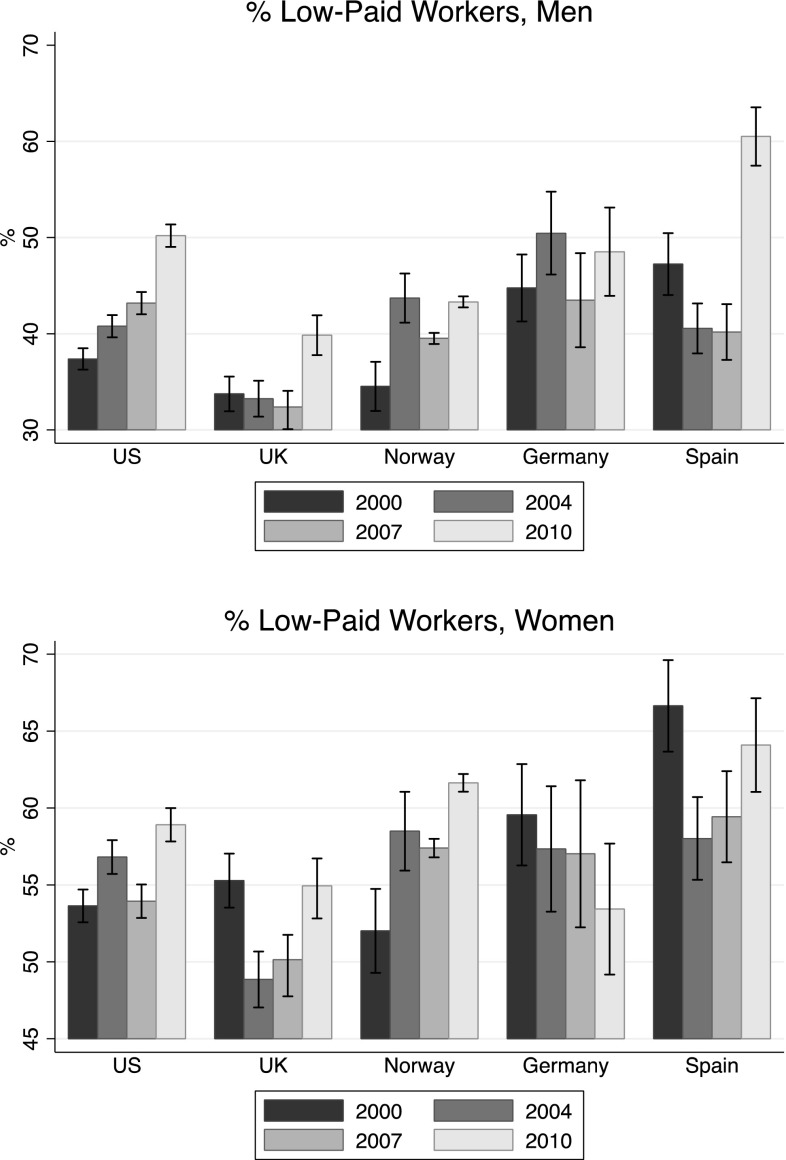
% of Men and women who are considered as *low-paid*

Figures 1 and 2 show the proportion of men and women working full-time and who were defined as *low-paid*, respectively. Generally, the proportion of young men working full-time decreased over time in all countries. The drop between 2007 and 2010—presumably due to the financial crisis—was quite substantial in the US (− 10.5%) and UK (− 10.2%), and even more in Spain (− 20%). The decrease was less evident in Norway (for which we report the proportion employed) and Germany. The trend was on a negative slope since the beginning of the twenty-first century, but the impact of the recession was very visible. Among young women, the situation was less clear-cut. Everywhere but in Spain the proportion of women working full-time was quite stable, and in the US, UK, and Norway, there was a 5–6% drop after the hit of the recession. In Germany, there was a 2% increase between 2007 and 2010. Spain showed a positive trend from 2000 to 2007—with an increase of almost 10%—but the crisis brought the proportion of women working full-time to a lower level than 2000 (40.3%).

If we look at the proportion of youth defined as *low-paid*, we observe a very similar trend among men and a quite clear impact of the crisis, especially in Spain, US and UK, where the proportion *low-paid* increased significantly between 2007 and 2010.

Among women, the trend over time was increasing in all the countries considered here, except for Germany, where there was a decrease from 59.6 to 53.4% between 2000 and 2010.

### What is Associated with Being Low-Paid?

**Fig. 3:**
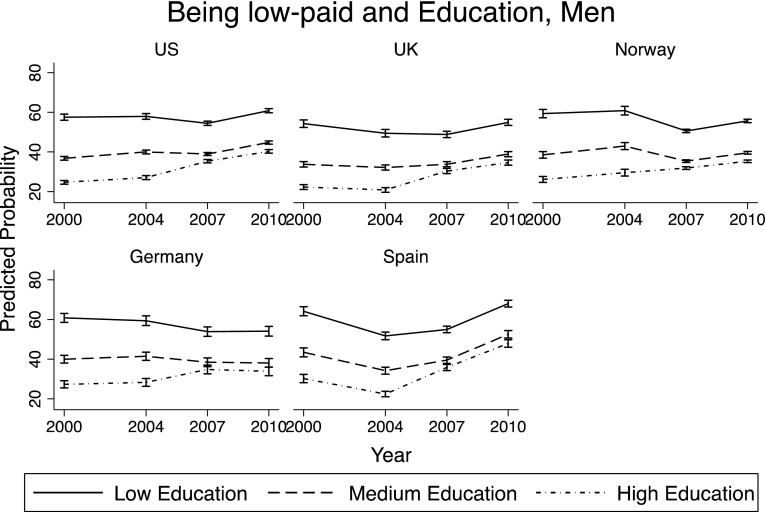
Predicted probability of being *low-paid*—men

**Fig. 4:**
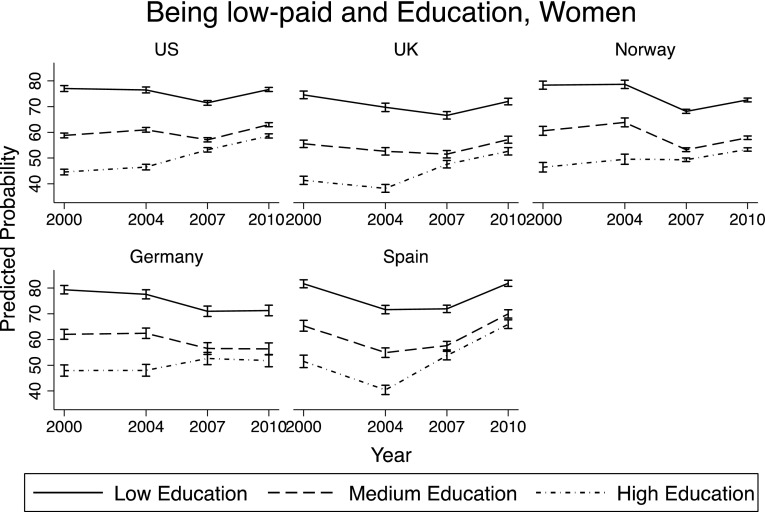
Predicted probability of being *low-paid*—women

In this section, the main factors associated with economic conditions of young adults were investigated. Through a set of logistic regressions,^4^ the predicted probability of being *low-paid* was computed depending on the year, country of residence, gender and level of education. The margins reported in Figs. 3 and 4 are based on a regression model that includes the following variables: age, gender, country, year, education, and interaction terms between year and gender, year and country variables, and year and education level.

Among men, the probability of being *low-paid* was always lowest among those with tertiary education, as expected. The levels were also quite comparable across countries. However, the trends over time differed across countries, and also showed a different impact of the economic recession. There was an increase in the predicted probability of being *low-paid* among young men starting in 2004 in US, UK, Germany, and Spain. In Norway this increasing trend started in 2000. There was an increase in the probability of being *low-paid* also for those with low and medium education (except than in Germany), especially after the crisis, but it is less pronounced than for those with high education. Very interestingly, across all countries, those who showed a larger increase in the probability of being *low-paid* between 2000 and 2010 were those with high education. This can be due to education expansion over this period of time. Moreover, the probability of being *low-paid* kept increasing between 2007 and 2010, which can be explained by the high populations of young men with tertiary education in the economic sectors most influenced by this crisis. However, it can also be due to the fact that these young men decided to stay longer in education (possibly going into graduate school), given the unfavorable conditions of the job market.

Figure 4 presents the results for young women: Probability of being *low-paid* is higher than for men in each education group, but the trends over time and the impact of the crisis are very similar across genders.

### Economic Conditions and Prolonged Education

As observed in Figs. 3 and 4, the group of young adults that showed the largest increase in the probability of being *low-paid* between 2000 and 2010 was that of highly educated men and women. Two explanations may be behind this result: On the one hand, it can be that high educated young employees were apart of the economic sectors with a declining performance over time and also got hit hardest by the crisis; on the other hand, it is also possible that young adults—given the lack of jobs—decided to stay in school longer and postpone their entry into the labor market. If including *enrollment in school* in the logistic regression makes the increase in the probability of being *low-paid* less marked (especially for those with a high level of education), it means that part of the increase in the probability of being low-paid over time is driven by young men and women staying in school longer, postponing the onset of financial stability.

**Fig. 5:**
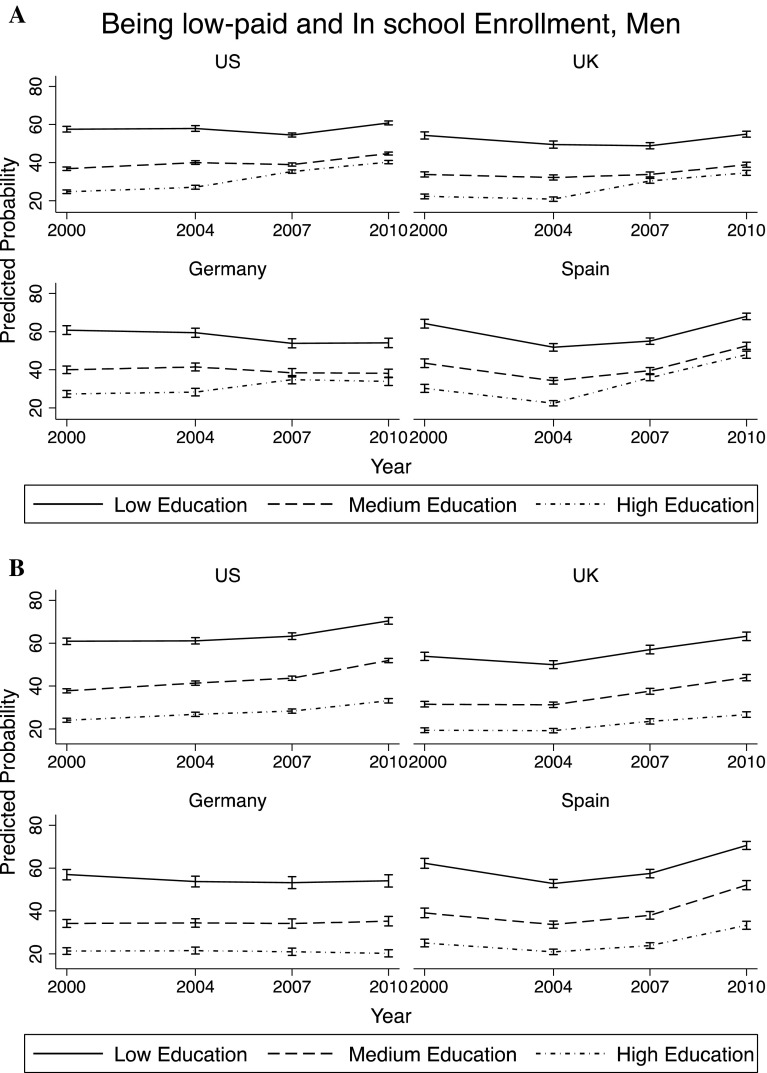
Being *low-paid* and enrollment in school—men. **a** Not controlling for *enrollment in school*. **b** Controlling for *enrollment in school*

**Fig. 6:**
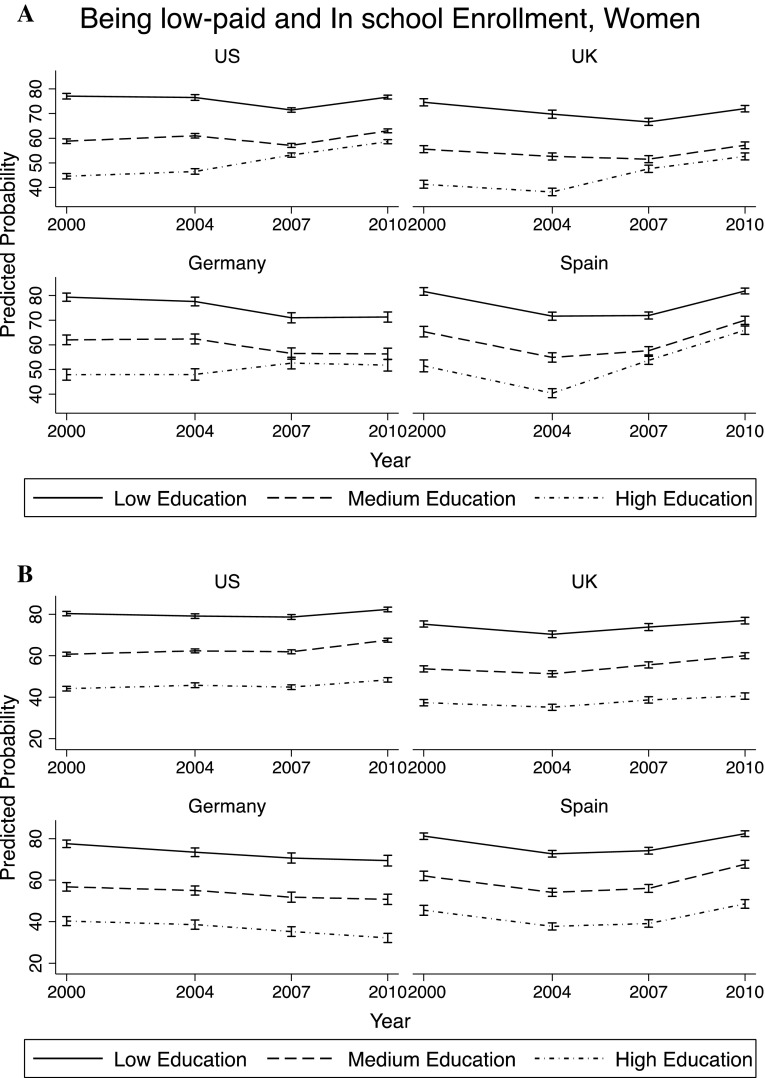
Being *low-paid* and enrollment in school—women. **a** Not controlling for *enrollment in school*. **b** Controlling for *enrollment in school*

The top part of Figs. 5 and 6 (part A) reports the analysis shown in Figs. 3 and 4 (without Norway, as there was no information on enrollment in school for 2000, 2004, and 2010),^5^ while the bottom part of Figs. 5 and 6 (part B) replicates the analysis including *enrollment in school*. It is very visible how in every country the increasing trend in the probability of being *low-paid* for high-educated young adults became much less pronounced. The changes were less marked for those with medium or low levels of education. This confirmed the hypothesis that the greater deterioration in financial conditions for those with high education between 2000 and 2010 was largely explained by their prolonged enrollment in school.

## Discussion

This article provides new evidence on the employment and economic conditions of young adults since 2000 and adopts a cross-national comparative perspective. Previous research on the effects of the recent economic crisis on labor market outcomes found an overall increase in unemployment rates, especially in youth unemployment rates (ILO 2010; Marcus and Gavrilovic 2010; O’Higgins 2014). This analysis confirms the previous findings by showing a substantial drop between 2007 and 2010 in the proportion of young men working full-time, a more modest decrease among women, and differences in magnitude across countries. The main contribution of this study is to look at the trends in income from work among young adults over time and across countries. This is important, given the increase in unemployment rate of youth, the increasing proportion of young adults who are NEETs (i.e., Not in Education, Employment, or Training), and the recent economic crisis. Young adults are less and less able to be financially stable, and monitoring the trends of income from work can help identify more vulnerable groups of people, and support policymakers’ decisions on welfare transfers. This work found that among men, the proportion of *low-paid* young adults has increased substantially in the US, the UK, and particularly in Spain. The increase has been smaller in Norway and almost non-existent in Germany. Similar trends were observed for women but with more variation over time and with a smaller increase due to the Great Recession. This could be due to the fact that more women had to start working during the crisis—to compensate for higher unemployment among men—and also to different fields of employment among women (e.g., service sectors) less hit by the crisis. These results are consistent with the predictions that can be derived by different welfare regimes. Where there is a generous welfare state (e.g., Norway) or where there is a strong vocational educational system (e.g., Germany), the increase over time and the impact of the recession on economic conditions of youth have been limited. Where there is a less generous welfare system—because of reliance on the economic market (e.g., US and UK), or on the family as the locus of support (e.g., Spain)—the increase over time and the damages of the crisis for young adults have been more visible. These results are particularly relevant for policy, because they imply that governments can moderate and intervene to reduce the negative impact of the crisis with welfare transfers. For women this trend is less evident, mainly because the female labor force participation rates have increased to a greater extent in the last five decades. But as women’s employment trajectories become more and more similar to those of men, it has to be expected that the role of the welfare regime will become the same across genders.

When looking at the predictors of economic position, there are not large differences across countries or across gender. What is important to notice is that the group that suffered the largest increase in the probability of being *low-paid* over time was the group with high education (i.e., tertiary education completed). This is primarily explained by the fact that young adults are reacting to the more competitive labor markets and to the financial crisis by staying in school longer and trying to protect themselves from economic uncertainty. An increase in the accumulation of human capital has been anticipated in the existing literature and it seems to be an important tool to increase future employment and income (Smeeding et al. 2011).

The presence of different welfare regimes in different countries partly explains some of the national differences and variation over time, especially relative to the influence of the crisis on economic position of young adults. However, some differences remain unexplained, and are possibly due to labor market regulations and cultural aspects. For example, in Southern European countries, where the age at leaving home is already higher than in other European and North American countries because of strong family ties, young adults facing a tighter economy may simply decide to live with their parents for longer. This means that they do not need to start working to provide for themselves, and this can partly explain the negative income trend observed over time.

The implications of these findings can also be important for other life domains. The uncertainty in the labor market, which causes higher unemployment rates and the postponement of economic stability among young adults, can contribute to shaping trends in family formation, fertility, and union dissolutions. The high increase in unemployment could directly influence the affordability of having children for some families. Moreover, rising unemployment is associated with a decrease—or postponement—in union formation (Prioux and Mandelbaum 2003). Divorce or union dissolution are also affected by the economic uncertainty, even though with less clear implications. Instability can increase both financial and psychological stresses, so it should, assumedly, also increase divorce rates. However, divorce is also expensive, so during times of crisis, couples may decide to stick together because they do not have enough resources to separate (Cherlin et al. 2013; Fischer and Liefbroer 2006; Sobotka et al. 2011).

The Luxembourg Income Study is a valuable source of information to compare individuals’ income across different countries, and to investigate long-term trends. However, this study suffers from several limitations that future research needs to address. First, it is necessary to look at how the generosity of different welfare regimes can affect the impact of the economic crisis. Social transfers can help mitigate earning disparities and attenuate the negative impacts of a financial crisis. New data should include governments’ transfers at the individual level in order to incorporate these transfers into the analysis of economic conditions of young adults. Moreover, it is also important to address, and formally test, the relevance of labor market regulations, or structure, and of culture for the employment and career trajectories of young adults. Also, when looking at economic conditions of youth, it would be ideal to have a more comprehensive measure of financial independence: This could be based on income from work, but also on many other factors, such as the cost of living (housing, food), public or private transfers, access to credit and future streams of income, and financial obligations. It should also take into account the family structure.

Finally, when more recent data will be available, it is necessary to look at the newest trends in young adults’ income from work to see whether there was recuperation after the Great Recession.

## Footnotes

1 1999 for the UK.

2 In the UK surveys only hours worked per week was included. The analysis looked only at full-time employment in order to link the analysis directly to economic conditions. Many young people worked in temporary or part-time positions, many were students (especially after the crisis). These workers would be defined as employed. However, part-time work is not conducive to financial stability, so it was not investigated in the analysis.

3 The highest completed level of education is coded into three categories: low (less than secondary education completed), medium (secondary education completed, completed ISCED levels 3 or 4), and high (tertiary education completed, ISCED levels 5 or 6).

4 Results of the regressions in tables format are available upon request.

5 The same results as in Figs. 5 and 6 were reported here again given that Norway was excluded from the analysis. This changed the dimension of the graphs and would prevent a clear comparability with the new analysis including *enrollment in school*.
